# Sleep duration is associated with liver steatosis in children depending on body adiposity

**DOI:** 10.1007/s00431-023-05332-2

**Published:** 2023-11-25

**Authors:** Begoña de Cuevillas, Judith Lubrecht, Santiago Navas-Carretero, Anita Vreugdenhil, J. Alfredo Martinez

**Affiliations:** 1 Center for Nutrition Research, Department of Nutrition, Food Sciences and Physiology, School of Pharmacy and Nutrition, University of Navarra, Irunlarrea 1, 31008 Pamplona, Spain; 2 Department of Pediatrics, Maastricht University Medical Centre, Maastricht, The Netherlands; 3 School of Nutrition and Translational Research in Metabolism (NUTRIM), Maastricht University, Maastricht, The Netherlands; 4 Centro de Investigación Biomédica en Red de la Fisiopatología de la Obesidad y Nutrición (CIBERobn), Instituto de Salud Carlos III, Madrid, Spain; 5 IdiSNA, Health Research Institute of Navarra, Pamplona, Spain; 6 Precision Nutrition Program, Research Institute On Food and Health Sciences IMDEA Food, CSIC-UAM, Madrid, Spain; 7 Centro de Medicina y Endocrinología, Universidad de Valladolid, Valladolid, Spain

**Keywords:** Obesity, Liver, Sleep, Interactions

## Abstract

Sleep is a factor associated with overweight/obesity risk, wherein interactions with fatty liver should be ascertained. The aim of this cross-sectional study was to analyze the possible relationships of sleep with liver health and whether this interplay is related to body adiposity distribution in children and adolescents. Anthropometric, clinical, and biochemical measurements were performed in children and adolescents (2–18 years old) with overweight/obesity (*n* = 854). Body fat distribution was clinically assessed, and several hepatic markers, including hepatic steatosis index, were calculated. Sleep time mediation (hours/day) in the relationship between the hepatic steatosis index and body fat distribution was investigated. Differences among diverse fatty liver disease scores were found between children with overweight or obesity (*p* < 0.05). Linear regression models showed associations between hepatic steatosis index and lifestyle markers (*p* < 0.001). Hepatic steatosis index was higher (about + 15%) in children with obesity compared to overweight (*p* < 0.001). Pear-shaped body fat distribution may seemingly play a more detrimental role on liver fat deposition. The association between sleep time and hepatic steatosis index was dependent on body mass index *z*-score. Post hoc analyses showed that 39% of the relationship of body fat distribution on hepatic steatosis index may be explained by sleep time.

*  Conclusion*: An association of sleep time in the relationship between body fat distribution and hepatic steatosis index was observed in children and adolescents with overweight/obesity, which can be relevant in the prevention and treatment of excessive adiposity between 2 and 18 years old. Clinical trial: NCT04805762.

*Import*: As part of a healthy lifestyle, sleep duration might be a modifiable factor in the management of fatty liver disease in children.

**Table:** 

**What is Known:** *• Sleep is an influential factor of overweight and obesity in children.* *• Excessive adiposity is associated with liver status in children and adolescents.*	
**What is New:** *• Sleep time plays a role in the relationship between body fat distribution and liver disease.* *• Monitoring sleep pattern may be beneficial in the treatment of hepatic steatosis in children with excessive body weight.*

**Supplementary Information:**

The online version contains supplementary material available at 10.1007/s00431-023-05332-2.

## Introduction

According to the World Health Organization (WHO), overweight and obesity are defined as abnormal or excessive body fat accumulation that often impairs health [1]. The increasing rates of persons with excessive weight-for-height are especially important in children and adolescents, as they are likely to evolve as adults with obesity and accompanying metabolic disturbances [2]. Indeed, overweight and obesity in children are associated with a higher risk of chronic disease incidences, such as cardiovascular diseases [3], type II diabetes [4], cancer [5], depression [6], or other mental disorders [7], and health impairments including sleep apnea later in life [8]. In Europe, nearly 14% of boys and 10% of girls aged from 7 to 8 years old had obesity [9].

Childhood management and obesity are individually influenced by multiple factors [10], such as environmental determinants, including physical (in)activity, screen time, (un)healthy eating behaviors, and personal features, but also genetics and gut microbiota composition [11], which should be monitored for implementation precision health and behavioral changes.

With the increasing prevalence of childhood obesity, non-alcoholic fatty liver disease (NAFLD), now renamed as metabolic dysfunction-associated steatotic liver disease (MASLD), has become the most prevalent cause of chronic liver disease in children and adolescents [12, 13]. Indeed, apple-shaped obesity has been more related to cardiovascular diseases than pear-shaped silhouettes [14], which has received scarce attention concerning this dimorphism in children with obesity. Thus, obesity is the main risk factor for MASLD [15], which is the most common cause of abnormal liver function in pediatric population [16, 17].

Different non-invasive tools and scores, relying on biochemical panels and/or imaging, have been studied in adults. These estimators are indirect proxies calculated from data that are routinely collected in children, which usually involved age, body mass index (BMI), and habitually available laboratory measurements such as triglycerides and transaminases [18].

In this context, sleep is an influential mediator in overweight and obesity conditions, which is receiving increasing attention [19]. Focused research indicates that inadequate sleep time and quality may contribute to fat depot enlargement and the development of overweight and obesity [20]. Moreover, increased sleep duration appears to elicit a positive influence on weight control in children and adolescents [21].

In this context, the aim of this ancillary research was to analyze the possible interaction of sleep duration with different liver markers and whether this interplay is related to underlying adiposity in children and adolescents.

## Material and methods

### Study design and population

The current data were derived from a longitudinal study conducted at the Centre for Overweight Adolescent and Children’s Healthcare (COACH) at the Maastricht University Medical Centre (MUMC +). COACH is a specialized center for the evaluation and treatment of children with overweight and obesity, which provides family-based lifestyle intervention to fight obesity in children and adolescents [22].

All children and adolescents with overweight or obesity, who participated in the COACH lifestyle intervention, were eligible for study inclusion. Overweight and obesity were defined according to the International Obesity Task Force (IOTF) criteria based on body mass index (BMI) [23]. A total of 854 children and adolescents, under 18 years of age, were identified as participants. Participants were classified according to sex, weight status, and developmental stage stratified by age as “early childhood” between 2 and 6 years old, “middle childhood” between 7 and 12 years old, and “adolescence” between 13 and 18 years old to screen youngster differences.

The study was conducted according to the Declaration of Helsinki and was approved by the Medical Ethical Committee of the MUMC + (METC 13–4-130, registered at ClinicalTrial.gov as NCT02091544). A signed informed consent from all necessary parties was obtained before inclusion in this study.

### Study measurements

Body weight was measured in underwear using calibrated electric scales (Seca© 877, Seca, Hamburg, Germany) to the nearest 0.1 kg. Standing height was measured using a rigid wall-based digital stadiometer (De Grood Metaaltechniek, Nijmegen, Netherlands) following standardized protocols.

Body mass index was calculated (BMI = weight [kg]/height [m]^2^). To correct for changes in BMI during childhood, age- and sex-specific BMI *z*-scores were extracted from the Growth Analyzer software (Growth Analyzer VE, Rotterdam, Netherlands) embedded in the electronic patient file. Children were classified as overweight: + 2 SD up to age 5, + 1 SD thereafter and obesity: + 3 SD up to 5 years, + 2 SD based on criteria of the IOTF, as described elsewhere [23].

Body circumferences were measured in standing position, using a non-elastic tape, while neck circumference was determined at the mid-thyroid level [22] by trained staff. Waist circumference (WC) was measured at the midpoint between the top of the iliac crest and the lower margin of the last palpable rib. Hip circumference was determined at the level of the maximum circumference of the gluteus. Thigh circumference was measured at the midpoint between the hip and knee, while the leg was bent in a 90° angle at the knee [22]. Body fat distribution was based on visual inspection by a clinician and subsequently classified as normal, pear, or apple-shaped body fat distribution. Waist-to-height ratio (WHtR) was calculated as a marker of adiposity distribution.

Blood pressure was measured about 20 times during a period of 1.5 h approximately to mitigate “white coat” interferences, in a sitting position using the Mobil-O-Graph equipment following the instructions of the supplier (IEM GmbH), where appropriate cuff size for the circumference of the upper arm was used as described for children [24]. Mean systolic blood pressure (SBP), diastolic blood pressure (DBP), and *z*-scores were calculated according to reference values related to height and sex [25]. Furthermore, blood biochemical markers were analyzed using validated standard operating procedures.

Lifestyle factors were collected through several questionnaires within a structured interview performed by a clinician. The questions conducted during the interview within a structured survey were as follows: How many hours a day do you sleep at night on a weekday?/How many glasses of sugary drinks do you consume in a day?/How many hours of screen time (TV, tablet, computer, etc.) do you watch in a day?/Do you do any physical exercise? Physical activity, dietary habits, and lifestyle factors were evaluated as published previously [21], which were ran under appropriate regression models.

### Hepatic markers

Different indirect hepatic markers have been calculated, considering the necessary criteria and applying accepted formulas [26]. The equations are shown in Supplementary Table 1. Hepatic steatosis index (HSI) was chosen as a proxy marker for hepatic steatosis in this young population for further analyses.

### Statistical analysis

A descriptive analysis concerning anthropometrics, biochemical, and hepatic markers across sex-, weight-, and age-specific groups was performed. The normality of the variables was screened using the Shapiro–Wilk test. Descriptive statistics were given as median and interquartile ranks (IQR), and differences were assessed by *t*-test or the Mann–Whitney test when non-normal distribution. Categorical variables were reported as percentages and compared with the chi-squared test. Some measurements and/or baseline data are missing, but apparently, these lacking data did not jeopardize outcomes when comparing “per protocol” and “intention-to-treat” approaches.

Lifestyle factors related to dietary and physical activity habits were chosen to construct the first linear regression model, which were adjusted for age, sleep time, sweet drink consumption, screen time, and insulin. This linear regression was not adjusted for variables such as sex and transaminases to avoid collinearity since these markers are within the equation to calculate HSI. The variance inflation factor (VIF) analysis for testing collinearity between independent variables ensured variable independence. Multiple linear regression models were used to predict liver damage, with HSI as proxy for liver disease. The variables used in the regression models were age, as it is a wide group; screen time, as a sedentary behavior variable; hours of sleep; consumption of sugar-sweetened beverages, as a dietary variable; insulin; as a proxy for health/glycemic status; and BMI and WC, as an anthropometric variable. Model 1 investigated the association between HSI and demographic characteristics and lifestyle factors including age (years), screen time (h/day), sleep time (h/day), sweet drinks consumption (glasses/day), and serum insulin levels (mU/L). Model 2 evaluated the association between HSI and age, sleep time, BMI *z*-score, and an interaction term between sleep time and BMI *z*-score. Mediation by sleep time in the relationship between liver damage (HSI) and body fat distribution in children and adolescents with overweight and obesity was further assessed using structural equation modeling following the Zhao et al*.* approach [27].

All *p*-values presented are two-tailed and were considered statistically significant at *p* < 0.05. Data were analyzed using STATA version 12.1 (StataCorp, College Station, TX, USA).

## Results

### Characteristics of the study population

The characteristics of the participants, separately analyzed by sex (*n* = 854), weight status (*n* = 843), and age (*n* = 842), including body composition, dietary, and lifestyle factors, are reported (Table 1). Anthropometric variables were significantly higher (*p* < 0.001) in older children and children with obesity, compared to younger children and children with overweight. Height (*p* = 0.0473), waist circumference (*p* = 0.0386), BMI *z*-score, and waist-to-hip ratio (WHr) (*p* < 0.001) were significantly higher in boys, while neck circumference was higher in girls (*p* < 0.001). Children with overweight and younger children (aged 3–6 years) slept significantly more hours per day during the workweek, compared to children with obesity (*p* = 0.0199) and older children (*p* = 0.007). Sports practice was significantly higher in children with obesity and children aged 7–12 years, compared to children with overweight (*p* < 0.001) and children aged 3–6 years and 13–18 years (*p* = 0.0022). Screen time was higher in girls (*p* = 0.0150) and children from 13 to 18 years old (*p* < 0.001).

**Table 1:** Children’s characteristics defined by sex, weight status, and development stage stratified by age as early childhood, middle childhood, and adolescence

	**Boys**	**Girls**	***p*-value**	**Overweight**	**Obese**	***p*-value**	**Early childhood (2–6 years old)**	**Middle childhood (7–12 years old)**	**Adolescence (13–18 years old)**	***p*-value**
	**400**	**454**		**181**	**662**		**69**	**428**	**345**	
General characteristics										
Age (years)	11.9 (9.8, 14.3)	12.1 (9.4, 15.5)	0.1287	11.8 (10.1, 14.0)	12.1 (9.5, 15.0)	0.5170	5.5 (4.5, 6.3)	10.6 (9.2, 11.7)	15.3 (14.2, 16.4)	** < 0.001**
Sex (%)			-			0.1701				0.981
Female	-	-		58.01	52.27		53.62	50.00	56.81	
Male	-	-		41.99	47.73		46.38	50.00	43.19	
Body composition										
Weight (kg)	68.0 (52.2, 89.9)	69.4 (48.1, 91.0)	0.6433	56.1 (46.2, 67.9)	72.2 (54.5, 95.8)	**< 0.001**	28.3 (25.9, 33.5)	57.2 (47.0, 69.0)	93.1 (80.7, 108.6)	**< 0.001**
Height (m)	1.6 (1.4, 1.7)	1.6 (1.4, 1.6)	**0.0473**	1.5 (1.4, 1.6)	1.6 (1.4, 1.7)	**0.0121**	1.2 (1.1, 1.2)	1.5 (1.4, 1.6)	1.7 (1.6, 1.7)	**< 0.001**
BMI (kg/m^2^)	28.0 (24.8, 32.7)	28.7 (24.5, 33.5)	0.5492	24.3 (22.5, 26.2)	30.1 (26.3, 34.6)	**< 0.001**	21.3 (20.4, 22.9)	26.3 (23.7, 29.3)	33.3 (29.5, 37.5)	**< 0.001**
BMI *z*-score	3.5 (3.0, 4.0)	3.1 (2.7, 3.6)	**< 0.001**	2.4 (2.2, 2.6)	3.5 (3.1, 4.0)	**< 0.001**	3.5 (2.8, 4.1)	3.1 (2.7, 3.6)	3.4 (2.9, 3.9)	**< 0.001**
Waist circumference (cm)	93.0 (82.5, 105.0)	89.2 (80.0, 102.8)	**0.0386**	82.0 (75.9, 88.0)	95.0 (85.0, 107.5)	**< 0.001**	68.0 (62.5, 72.2)	86.4 (79.0, 94.0)	104.0 (93.1, 112.7)	**< 0.001**
Waist circumference *z*-score	5.4 (4.1, 7.2)	4.8 (3.7, 6.7)	**0.0016**	3.5 (2.6, 4.3)	5.7 (4.4, 7.5)	**< 0.001**	3.7 (2.8, 4.5)	4.4 (3.4, 5.6)	6.9 (5.1, 8.1)	**< 0.001**
Hip circumference (cm)	99.0 (88.8, 10.5)	103.0 (87.0, 116.5)	0.0514	91.4 (83.0, 99.3)	105.0 (92.0, 117.3)	**< 0.001**	72.1 (68.0, 78.0)	94.0 (85.0, 101.0)	114.5 (106.0, 123.0)	**< 0.001**
Waist-to-hip ratio (WHr)	0.9 (0.9, 1.0)	0.9 (0.8, 1.0)	**< 0.001**	0.9 (0.8, 1.0)	0.9 (0.9, 1.0)	**< 0.001**	0.9 (0.9, 1.0)	0.9 (0.9, 1.0)	0.9 (0.8, 1.0)	**< 0.001**
Neck circumference (cm)	34.0 (31.1, 36.8)	35.0 (32.5, 38.0)	**< 0.001**	32.5 (30.5, 34.5)	35.1 (32.5, 38.3)	**< 0.001**	29.5 (27.5, 30.5)	33.0 (31.3, 35.0)	37.3 (35.0, 40.1)	**< 0.001**
Thigh circumference (cm)	55.0 (48.0, 62.3)	53.2 (47.8, 59.9)	0.1125	50.0 (45.0, 54.0)	57.0 (49.8, 63.6)	**< 0.001**	40.0 (37.0, 43.3)	50.5 (47.0, 55.0)	62.0 (57.5, 67.6)	**< 0.001**
Body fat distribution (%)			0.7837			**0.0025**				**0.0296**
Normal	56.7	61.7		69.8	56.4		66.2	60.6	56.2	
Apple	41.6	27.6		24.9	36.9		33.8	37.0	31.1	
Pear	1.7	10.7		5.3	6.7		0.0	2.4	12.7	
Lifestyle factors										
Sleep time during week (hours/day)	9.5 (8.5, 10.4)	9.5 (8.5, 10.5)	0.6965	10.0 (9.0, 10.5)	9.5 (8.0, 10.5)	**0.0199**	11.0 (10.5, 11.5)	10.0 (9.5, 10.5)	8.25 (7.5, 9.0)	**< 0.001**
Sweet drinks (glasses/day)	2.0 (1.0, 4.0)	2.0 (1.0, 4.0)	0.6395	2.0 (0.5, 3.0)	2.0 (1.0, 4.0)	0.1243	1.5 (0.5, 3.0)	2.0 (1.0, 4.0)	2.0 (1.0, 4.0)	0.1293
Screen time (hours/day)	3.0 (2.0, 4.0)	3.0 (1.5, 4.0)	**0.0150**	3.0 (1.8, 4.0)	3.0 (2.0, 4.5)	0.2617	1.5 (1.0, 2.0)	2.5 (1.5, 4.0)	4.0 (2.5, 5.0)	**< 0.001**
Sport (%)	46.7	53.3	0.8414	25.5	74.1	**0.0007**	7.6	55.6	36.8	**0.0022**

Variables are shown as median (IQR) according to its distribution. *BMI*, body mass index. BMI *z*-score was estimated with Cole et al. formula. Discrepancies in the sample size are due to missing data for some participants on some parameters

Clinical and biochemical measurements are reported (Table 2) where fasting plasma glucose (*p* = 0.0170) and CRP (*p* = 0.0289) were significantly elevated in boys compared to girls. Also, SBP (*p* < 0.001), HOMA-IR (*p* = 0.0133), CRP (*p* < 0.001), LDL-c (*p* = 0.0157), and GGT (*p* = 0.0277) were significantly higher in children with obesity than in children with overweight, while HDL-c (*p* < 0.001) concentrations were higher in children with overweight than in children with obesity. On the other hand, SBP (*p* < 0.001), DBP (*p* < 0.001), HOMA-IR (*p* < 0.001), TG (*p* < 0.001), TyG (*p* < 0.001), and CRP (*p* = 0.0015) were significantly different between age categories, with the highest values found in children aged 13–18 years than in children under 13 years old (Table 2). Contrarywise, HDL-c (*p* < 0.001) was significantly higher in children aged 3–6 years than in children from 7 to 18 years old.

**Table 2:** Children’s clinical and blood biochemical markers characterized by sex, weight status, and development stage stratified by age as early childhood, middle childhood, and adolescence

	**Boys**	**Girls**	***p*-value**	**Overweight**	**Obese**	***p*-value**	**Early childhood (2–6 years old)**	**Middle childhood (7–12 years old)**	**Adolescence (13–18 years old)**	***p*-value**
***n***	**400**	**454**		**181**	**662**		**69**	**428**	**345**	
Clinical and blood biochemical markers										
SBP (mmHg)	116.0 (110.0, 124.0)	117.0 (111.0, 123.0)	0.7720	113.0 (108.0, 121.0)	118.0 (111.9, 124.0)	**< 0.001**	110.0 (102.5, 115.5)	114.0 (109.0, 120.0)	120.0 (114.0, 128.0)	**< 0.001**
DBP (mmHg)	68.0 (63.0, 72.0)	68.0 (63.0, 73.3)	0.1334	68.0 (62.0, 72.0)	68.0 (63.0, 73.0)	0.0948	64.5 (60.0, 69.0)	67.0 (62.0, 71.0)	70.0 (65.0, 75.0)	**< 0.001**
Glucose (mg/dl)	79.3 (72.1, 88.3)	77.5 (70.3, 86.5)	**0.0170**	81.1 (72.1, 88.3)	77.5 (70.3, 86.5)	0.1327	80.2 (73.9, 88.3)	79.3 (71.2, 88.3)	77.5 (72.1, 86.5)	0.6119
HOMA-IR	2.1 (0.7, 3.9)	2.4 (1.0, 4.0)	0.2110	1.8 (1.0, 3.2)	2.4 (0.8, 4.2)	**0.0133**	0.4 (0.0, 1.6)	1.9 (0.8, 3.6)	2.9 (1.7, 5.0)	**< 0.001**
Total cholesterol (mg/dl)	158.3 (139.0, 177.6)	162.2 (142.0, 185.3)	0.1601	158.3 (142.9, 175.7)	162.2 (142.9, 181.5)	0.2215	158.3 (142.9, 173.7)	162.2 (142.9, 185.3)	158.3 (139.0, 177.6)	0.1078
LDL-c (mg/dl)	92.7 (73.4, 108.1)	92.7 (77.2, 112.0)	0.3255	88.8 (73.4, 108.1)	92.7 (77.2, 112.0)	**0.0157**	88.8 (81.1, 104.2)	92.7 (77.2, 112.0)	88.8 (73.4, 112.0)	0.3437
HDL-c (mg/dl)	46.3 (42.5, 54.1)	46.2 (42.5, 54.1)	0.7123	50.2 (42.5, 57.9)	46.3 (38.6, 54.1)	** < 0.001**	54.1 (46.3, 61.8)	50.2 (42.5, 57.9)	46.3 (38.6, 54.1)	**< 0.001**
TG (mgdl)	85.0 (60.2, 115.0)	88.5 (64.6, 119.5)	0.1792	84.8 (58.0, 111.1)	86.7 (63.7, 119.5)	0.1004	62.8 (46.0, 86.7)	87.6 (61.9, 115.9)	89.8 (66.4, 126.5)	**< 0.001**
TyG (md/dl)	8.1 (7.8, 8.5)	8.1 (7.8, 8.5)	0.5968	8.1 (7.7, 8.4)	8.1 (8.0, 8.5)	0.3111	7.8 (7.5, 8.2)	8.1 (7.8, 8.5)	8.2 (7.8, 8.5)	**< 0.001**
ALT (U/L)	23.0 (21.0, 30.0)	22.0 (17.0, 30.0)	0.4919	22.0 (18.0, 29.5)	22.0 (17.0, 30.0)	0.7535	22.0 (17.0, 28.0)	22.0 (17.0, 30.0)	22.0 (18.0, 31.0)	0.7697
AST (U/L)	25.0 (21.0, 31.0)	26.0 (20.0, 32.0)	0.7854	26.0 (21.0, 32.0)	26.0 (20.0, 31.0)	0.2466	26.5 (22.0, 33.0)	25.0 (20.0, 31.0)	26.0 (21.0, 32.0)	0.3368
GGT (U/L)	16.0 (13.0, 21.0)	17.0 (13.0, 21.0)	0.9156	15.0 (12.0, 20.0)	17.0 (13.0, 21.0)	**0.0277**	15.0 (12.0, 20.0)	16.0 (13.0, 21.0)	17.0 (13.0, 21.0)	0.1649
Insulin (mU/L)	15.3 (8.9, 22.7)	13.5 (8.1, 21.7)	0.0843	12.7 (6.9, 21.7)	14.6 (8.9, 22.2)	0.0684	11.5 (6.9, 17.8)	14.4 (8.9, 23.3)	14.6 (8.4, 21.7)	0.0788
CRP (mg/L)	2.0 (1.0, 5.0)	2.0 (2.0, 5.0)	**0.0289**	1.0 (1.0, 3.0)	2.0 (1.0, 5.0)	**< 0.001**	2.0 (1.0, 3.0)	2.0 (1.0, 4.0)	2.0 (1.0, 6.0)	**0.0015**
HbA1C (%)	5.2 (5.0, 5.5)	5.3 (5.0, 5.5)	0.1055	5.2 (5.0, 5.5)	5.2 (5.0, 5.4)	0.3029	5.2 (4.9, 5.4)	5.2 (5.0, 5.5)	5.2 (5.0, 5.5)	0.3771
Platelets (10^9^/L)	310 (267, 352)	302(261, 348)	0.6871	312 (262, 349)	302 (263, 351)	0.6068	324.5 (269, 353.5)	306 (261, 353)	300 (264, 345.5)	0.7286
FFA (mmol/L)	0.7 (0.5, 0.8)	0.7 (0.5, 0.8)	0.5817	0.7 (0.5, 0.9)	0.7 (0.5, 0.8)	0.2034	0.7 (0.5, 0.9)	0.7 (0.5, 0.8)	0.7 (0.5, 0.8)	0.9645
Alkaline phosphatase (U/L)	233.5 (147.0, 303.0)	237.0 (139.0, 304.5)	0.9971	241.0 (143.0, 302.0)	235.0 (146.0, 304.0)	0.7792	243.5 (152.0, 315.0)	232.0 (139.0, 300.0)	235.0 (140.0, 306.0)	0.7590
Bilirubin (µmol/L)	6.4 (4.7, 8.9)	6.7 (4.9, 9.3)	0.2255	6.6 (4.9, 8.9)	6.5 (4.7, 9.2)	0.5293	6.0 (4.2, 8.2)	6.6 (4.9, 9.4)	6.4 (4.8, 9.2)	0.2308

Variables are shown as median (IQR) according to its distribution. Discrepancies in the sample size are due to missing data for some participants on some parameters.

*SBP* systolic blood pressure, *DBP* diastolic blood pressure, *HOMA-IR* homeostasis model assessment-insulin resistance, *LDL-c* low-density lipoprotein cholesterol, *HDL-c* high-density lipoprotein cholesterol, *TG* triglycerides, *TyG* triglyceride glucose index, *ALT* alanine aminotransaminase, *AST* aspartate aminotransaminase, *GGT* gamma-glutamyl transferase, *CRP* c-reactive protein, *FFA* free fatty acids

### Hepatic markers

Means comparison between children with overweight and obesity revealed differences in most of the assessed hepatic indices (Table 3). Fatty liver index (FLI), WHtR, WC*TyG, HSI, lipid accumulation product (LAP), Zhejiang University Index (ZJU Index), FibroMeter, and pediatric metabolic index (PMI) were found to be statistically higher in children with obesity (*p* < 0.001), compared to children with overweight (Table 3). Furthermore, visceral adiposity index (VAI) and NAFLD fibrosis score (NFS) showed a marginal trend towards statistical significance, increasing in VAI and decreasing in NFS (*p* = 0.053 and *p* = 0.063, respectively).

**Table 3:** Comparison of children’s adiposity, metabolic, and hepatic markers according to their body weight

	**Overweight**	**Obese**	***p*-value**
***n***	**181**	**662**	
Hepatic markers			
Waist-to-height ratio (WHtR)	0.5 (0.5, 0.6)	0.6 (0.6, 0.7)	**< 0.001**
Visceral adiposity index (VAI)	2.5 (1.6, 3.9)	2.9 (1.8, 4.6)	0.0053
Triglyceride-glucose index (TyG)	8.1 (7.7, 8.4)	8.1 (8.0, 8.5)	0.3111
Hypertriglyceridemic-waist index (WC*TyG)	660.9 (603.3, 734.7)	771.3 (681.9, 881.4)	**< 0.001**
Lipid accumulation product (LAP)	17.6 (9.8, 30.1)	30.5 (16.6, 53.8)	**< 0.001**
Fatty liver index (FLI)	0.5 (0.3, 0.9)	2.1 (0.7, 7.5)	**< 0.001**
Hepatic steatosis index (HSI)	34.1 (32.3, 36.2)	40.0 (35.9, 44.6)	**< 0.001**
Zhejiang University Index (ZJU)	33.8 (31.4, 36.2)	39.4 (35.3, 44.4)	**< 0.001**
Fibrosis-4 (FIB-4)	0.2 (0.2, 0.3)	0.2 (0.1, 0.3)	0.5963
AST to platelet ratio index (APRI)	0.2 (0.2, 0.3)	0.2 (0.2, 0.3)	0.1443
FORNS index	−1.5 (−2.4, −0.8)	−1.5 (−2.5, −0.5)	0.5663
NAFLD fibrosis score (NFS)	−28.8 (−30.2, −27.2)	−28.2 (−30.0, −26.5)	0.0633
FibroMeter	18.0 (17.1, 18.7)	18.7 (17.5, 20.1)	**< 0.001**
Pediatric NAFLD Fibrosis Index (PNFI)	2.5 (0.6, 5.8)	1.8 (0.3, 5.7)	0.2731
Pediatric NAFLD Fibrosis Score (PNFS)	31.6 (25.8, 37.9)	30.6 (24.4, 37.6)	0.3162
Pediatric metabolic index (PMI)	0.1 (0.1, 0.1)	0.2 (0.1, 0.2)	**< 0.001**

Variables are shown as median (IQR) according to its distribution

### Relationship between liver damage and lifestyle factors

**Fig. 1:**
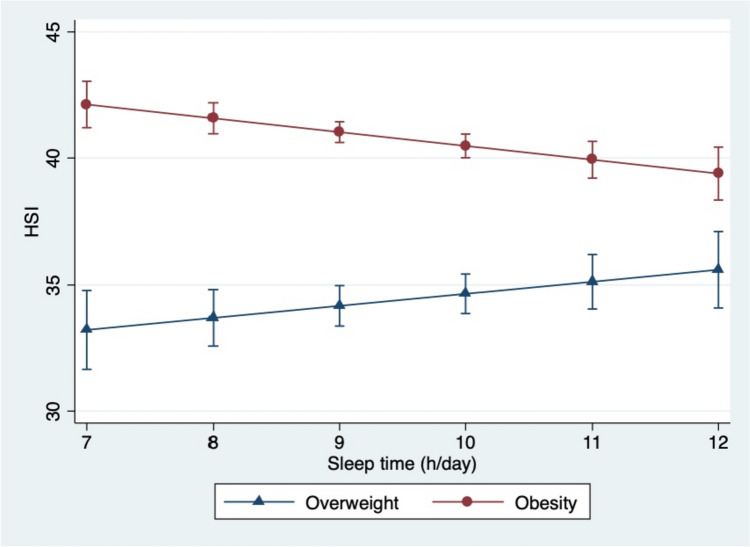
Effect of the changes in HSI and sleep time and body weight categories in children and adolescents with overweight and obesity

A significant inverse correlation was found between HSI and sleep time (*β* = 0.437, *p* = 0.027), while a positive association existed between HSI and sweet drinks consumption (*β* = 0.180, *p* = 0.045) and screen time (*β* = 0.506, *p* = 0.002) as reported (Table 4). No significant association was found for insulin (*p* = 0.945). The second regression model showed a significant interaction (Table 4) between sleep time in hours and BMI *z*-score (*p* = 0.001). Change in HSI by hours of sleep is plotted (Fig. 1). This figure is a mathematical model showing that HSI decreases in children with obesity (BMI *z*-score > 3), as hours of sleep per day increase, while in children with overweight (BMI *z*-score between 2 and 3), HSI remains stable, regardless of sleep time, and below of the HSI cut-off (Fig. 1). This result shows that the higher the weight status, the more influence sleep hours have on liver HSI. Youngsters with obesity showed higher HSI in both apple- and pear-shape than overweight children. However, differences were only found between HSI values in boys and girls in the apple-shape group, but not in the pear-shape group.

**Table 4:** Linear regression model of liver damage based on the HSI index as dependent variable

	**Liver damage (HSI)**		
	***β***	***p*-value**	**R**^**2**^
Model 1		**< 0.001**	0.4076
Age (years)	1.064	**< 0.001**	
Screen time (h/day)	0.406	**0.002**	
Sleep time (h/day)	−0.437	**0.027**	
Sweet drinks consumption (glasses/day)	0.180	**0.045**	
Insulin (mU/L)	−0.001	0.945	
Model 2		**< 0.001**	0.7864
Age (years)	0.498	**< 0.001**	
Waist circumference (cm)	0.198	**< 0.001**	
WHR	−15.280	**< 0.001**	
Sleep time (h/day)	2.804	**< 0.001**	
BMI *z*-score	11.627	**< 0.001**	
Sleep time # BMI *z*-score	−0.846	**< 0.001**	

*β* represents changes in outcomes for the increasing number of units of HSI in the population. Bold numbers indicate statistical significance (*p* < 0.05)

### Mediation model concerning HSI outcomes

**Fig. 2:**
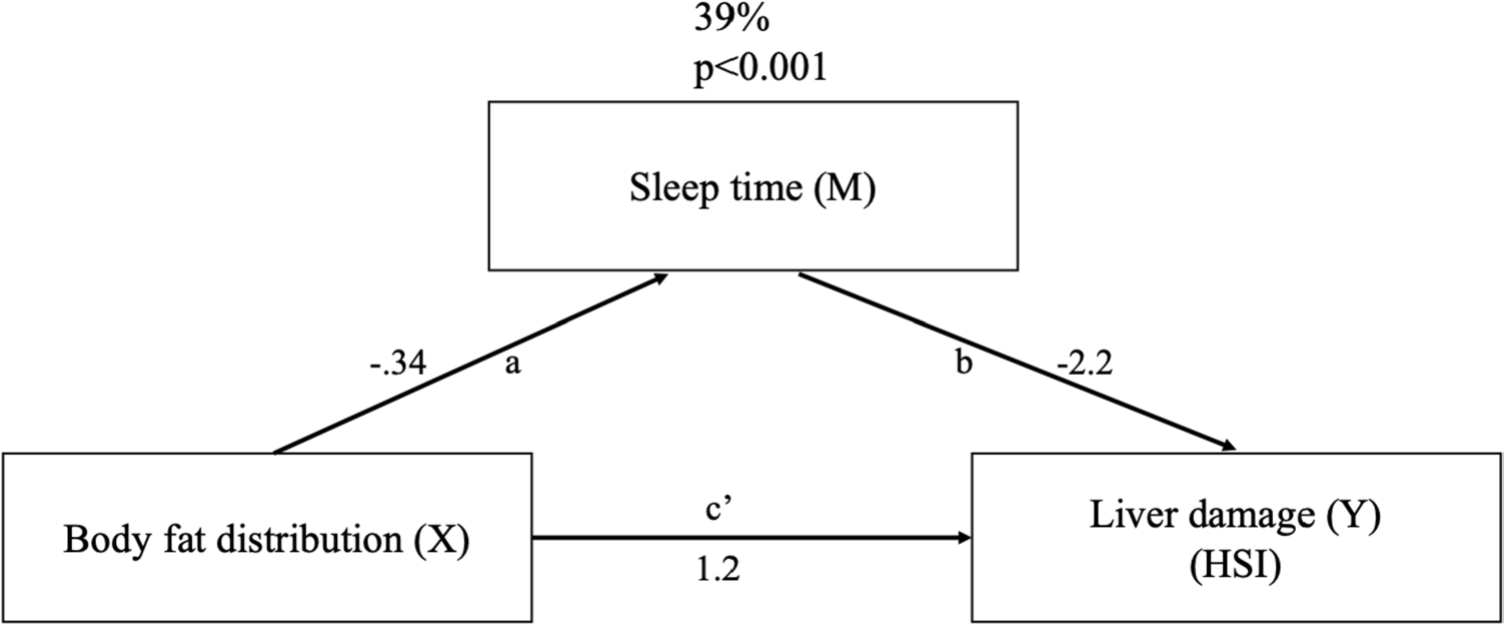
Diagram of the simple mediation model, concerning sleep time-mediated relationship between body fat distribution and HSI in children with overweight and obesity. The paths of the model labeled *a*, *b*, and *c’* are estimated using the 3 regressions equations represented in the diagram. The indirect or mediated effect of *X* or *Y* through *M* (39%) is quantified from *a* and *b* values

Children with obesity showed a higher HSI (34.1 vs 40.0) as compared to children with overweight (*p* < 0.001), while girls evidenced a higher (*p* < 0.001) HSI than boys (39.4 vs 37.0). In this study, a pear-shaped body fat distribution was found to be more detrimental (*p* < 0.001) concerning the development of liver steatosis (HSI = 44.1; CI, 40.6;48.2) as compared to normal distribution (HSI = 37.3; CI, 34.1; 41.7) with no differences between apple silhouette and normal distribution. An effect modification by sleep time in the relationship between body fat distribution and HSI index was found in children and adolescents (Fig. 2). The mediation model of body fat distribution influencing HSI showed an interactive association of sleep duration in this relationship, indicating that 39% of the association of body fat distribution on liver steatosis (HSI index) is mediated by sleep time per day. The mediation model showed an association between sleep time and body composition, which in turn is related to liver health.

## Discussion

To the best of our knowledge, this study is a pioneer in investigating the relationship of sleep duration on liver damage in children with overweight and obesity evidencing an inverse association between liver disease measured through HSI and sleep duration. The relationship between sleep duration and HSI is mediated by the BMI *z*-score. Pear-shaped body fat distribution was found to be more detrimental for the development of liver steatosis than normal or apple distribution when assessed via HSI.

Children with obesity showed higher HSI in both apple and pear shapes than children with overweight. Only differences between HSI values in children were found in the apple-shape group, but not in the pear-silhouette group, which should be interpreted with caution, as abdominal obesity is usually more associated with cardiovascular diseases than gluteus-femoral adiposity in adults [14].

Interestingly, those children and adolescents with higher HSI as an indicator of more severe steatosis elicited better reductional outcomes when increasing sleep time (hours/day), which is a pattern commonly found in metabolic diseases [28]. However, Biemans et al. [29] found that sleep quality of children with chronic metabolic conditions is similar to the normal children group. Obstructive sleep apnea is a prevalent disorder in children that is related to sleep duration and is associated with metabolic, cardiovascular, and neurocognitive morbidities [30]. Indeed, a bidirectional association between obstructive sleep apnea and MASLD has been reported [31].

Previous studies have linked BMI to sleep duration in children, with subjects who sleep less, having a higher BMI [28, 32]. In fact, some researchers [33] established that sleep duration is inversely correlated with the risk of excessive body weight. In adults, sleep has been associated with MASLD development with or without fibrosis [34]. Short sleep duration and poor sleep quality increased the risk of fatty liver disease [35, 36]. Carotenuto et al*.* [37] demonstrated a strong relationship between obstructive sleep apnea and liver damage in pediatric MASLD, but there is no information about sleep duration. Our study showed an inverse association between sleep hours and liver damage.

Body fat distribution has been suggested as a key point for the onset of liver damage, while BMI has previously been inversely linked with sleep duration. Indeed, apple and pear body type as defined by subjective visual inspection in clinical practice has been also implemented in research to characterize obesity [38]. About 39% of the association of body fat distribution on liver steatosis (HSI index) is mediated by sleep duration. These results are supported in adults, since Imaizumi et al*.* [39] found that short sleep duration tends to be associated with MASLD which may be mediated by abdominal body adiposity in adult women.

Interestingly, a significant relationship was found between liver damage as measured through HSI and adjustment variables such as screen time and sugar sweetener beverage consumption consistent with previous scientific literature [40–47]. No relationship between insulin and HSI as a liver damage proxy was found, as other investigators reported [48–50].

As expected, there were differences in specific clinical and biochemical markers between age groups, sexes, and different IOTF groups [51]. Similar to our study, Higgins et al*.* [52] observed lower HDL-c levels in subjects with obesity in comparison to subjects with overweight. The observed differences between age group results are in line with previous investigations that evidenced a relationship between age and clinical and biochemical markers such as blood pressure [53], serum lipid measurements [54], and HOMA-IR [55], even at early life stages [56]. It would be valuable to conduct an exhaustive analysis about this issue between ages in subsequent studies.

In our study, we found that several noninvasive hepatic scores, which assessed different aspect and morbid features [26], appeared to be related to obesity in youngsters. Some indices have not been validated in children and might not be suitable for detecting and monitoring MASLD in children, but they may provide valuable information on different aspects of liver disease, such as composition, function, and lipid metabolism in clinical practice [57]. In any case, HSI has been implemented in the pediatric population [58].

### Strengths and limitations

This research was the first to investigate the relationship of sleep duration on liver damage through indirect indexes in children with overweight and obesity. The data arising from this study may be of significant value in the treatment of liver disease in pediatric practice. A relationship between sleep, body composition, and MASLD has also been established, although it cannot be ruled out that other factors may be involved in this mediation. Due to the study design, and the scarcity of related literature, these findings should be treated with caution. The potential low validity of some hepatic markers in children and adolescents is a limitation to be accounted for, while the shape (apple vs pear) may be a discriminating prognostic factor. To discard hypertension or “white coat” interferences, blood pressure should be collected in a 24-h monitor, which should be mentioned as a limitation of the study, despite making 20 blood pressure measurements with this objective.

## Conclusions

This research shows that sleep time plays an interactive role in the relationship between body fat distribution and liver disease. In fact, 39% of the impact of body fat distribution on HSI was influenced by sleep duration in children with overweight and obesity. These results determine that monitoring and advising a healthy sleep pattern may prove beneficial and suitable in the management of liver steatosis in children and adolescents with overweight and obesity.

### Supplementary Information

Below is the link to the electronic supplementary material.

Supplementary file1 (DOCX 19 KB)

## Data Availability

The datasets generated and analyzed during this study concerning the COACH cohort (METC 13–4-130) are not publicly available as some participants in this study did not provide consent to do so. Data can be made available from author A.C.E. Vreugdenhil through data sharing agreements at reasonable request. On the other hand, related information about this ongoing cohort can be found in 10.1210/jc.2015-1444 and will be part of the PhD dissertation of Judith Lubrecht. This study is registered at ClinicalTrial.gov (NCT02091544).
