# Supplementary material for: Sleep duration is associated with liver steatosis in children depending on body adiposity
Source: Eur J Pediatr. 2023 Nov 25;183(2):779–89. doi: 10.1007/s00431-023-05332-2 (PMC10912132; doi:10.1007/s00431-023-05332-2)
Supplement: Supplementary file 1 — Supplementary file1 (DOCX 19 KB) [file 431_2023_5332_MOESM1_ESM.docx]

**Supplemental table 1**. Equations for the calculation of the different hepatic indexes.

| **Index** | **Equation** |
| --- | --- |
| Waist to Height Ratio (WHtR) | $WHtR=\frac{waist circumference}{\mathrm{height}}$ |
| Visceral Adiposity Index (VAI) | VAI (men) = [$\frac{\mathrm{WC}}{39.68}$+(1.88*BMI)]*$\frac{\mathrm{TG}}{1.03}*\frac{1.31}{\mathrm{HDL}-c}$ |
|  | VAI (women) = [$\frac{\mathrm{WC}}{36.58}$+(1.89*BMI)]*$\frac{\mathrm{TG}}{0.81}*\frac{1.52}{\mathrm{HDL}-c}$ |
| TyG Index | $TyG=ln\frac{TG*Glucose}{2}$ |
| WC*TyG | WC*TyG = WC*TyG |
| Lipid Accumulation Product (LAP) | $\mathrm{LAP}\left( \mathrm{men} \right)=\left( WC-65 \right)*TG$ |
|  | $\mathrm{LAP}\left( \mathrm{women} \right)=\left( WC-58 \right)*TG$ |
| Fatty Liver Index (FLI) | $FLI=\frac{e^{0.953*loge\left( \mathrm{TG} \right)+0.139*BMI+0.178*loge\left( \mathrm{GGT} \right)+0.053*WC-15.745}}{1+e^{0.953*loge\left( \mathrm{TG} \right)+0.139*BMI+0.178*loge\left( \mathrm{GGT} \right)+0.053*WC-15.745}}*100$ |
| Hepatic Steatosis Index (HSI) | $HSI=8*\frac{\mathrm{ALT}}{\mathrm{AST}}+BMI (+2 if T2D,+2 if female)$ |
| Zhejian University (ZJU) Index | $ZJU=BMI+FPG+TG+3*\frac{\mathrm{ALT}}{\mathrm{AST}}(+2 if female)$ |
| Fibrosis-4 (FIB-4) | $FIB-4=\frac{Age*AST}{Platelets*\sqrt{\mathrm{ALT}}}$ |
| AST to Platelet Ratio Index (APRI) | $APRI=\frac{\frac{\mathrm{AST}}{\mathrm{ULN}}}{\mathrm{Platelets}}*100$ |
| FORNS Index | $FORNS=7.811-3.131*\ln platelets+0.781*\ln\mathrm{GGT}+3.467*\ln\mathrm{age}-0.014*cholesterol$ |
| NAFLD Fibrosis Score (NFS) | $NFS=-1.675+0.037*Age+0.094*BMI+1.13*IFGorT2D(yes:1,no:0)+0.99*\frac{\mathrm{AST}}{\mathrm{ALT}}-0.013*platelets-0.66*albumin$ |
| Fibrometer | P=0.4184*glucose+0.0701*AST+0.0008*ferritin-0.0102*platelets-0.0260*ALT+0.0459*body weight+0.0842*age+11.6226 |
| Pediatric NAFLD Fibrosis Index (PNFI) | $PNFI=\frac{1}{1+e^{-(-6.539*loge\left( \mathrm{age} \right)+0.207*WC+1.957*loge(TG)-10.074}}*10$ |
| Pediatric NAFLD Fibrosis Score (PNFS) | PNFS=$\frac{e^{1.1*\left( 0.34+\sqrt{\mathrm{ALT}} \right)+\left( 0.002*ALP \right)-\left[ 1.1*\log\left( \mathrm{platelets} \right) \right]-(0.02*GGT)}}{1+e^{1.1*\left( 0.34+\sqrt{\mathrm{ALT}} \right)+\left( 0.002*ALP \right)-\left[ 1.1*\log\left( \mathrm{platelets} \right) \right]-(0.02*GGT)}}*100$ |
| Pediatric Metabolic Index (PMI) | PMI (men < 10 years old) = $\frac{\mathrm{WC}}{(-0.02\mathrm{BMI}^{2}+3.62BMI+3.72)}*\frac{\mathrm{TG}}{0.77}*\frac{1.38}{\mathrm{HDL}-c}$ |
|  | PMI (men ≥ 10 years old) = $\frac{\mathrm{WC}}{(-0.02\mathrm{BMI}^{2}+3.62BMI+3.72)}*\frac{\mathrm{TG}}{1.06}*\frac{1.30}{\mathrm{HDL}-c}$ |
|  | PMI (women < 10 years old) = $\frac{\mathrm{WC}}{(-0.02\mathrm{BMI}^{2}+3.67BMI+3.24)}*\frac{\mathrm{TG}}{0.88}*\frac{1.32}{\mathrm{HDL}-c}$ |
|  | PMI (women ≥ 10 years old) = $\frac{\mathrm{WC}}{(-0.02\mathrm{BMI}^{2}+3.67BMI+3.24)}*\frac{\mathrm{TG}}{1.04}*\frac{1.34}{\mathrm{HDL}-c}$ |

WHtR, Waist to Height Ratio; VAI, Visceral Adiposity Index; WC, Waist Circumference; BMI, Body Mass Index; TG, Triglycerides; HDL-c, High-Density Lipoprotein Cholesterol; TyG, Triglyceride-Glucose Index; WC*TyG, Hypertriglyceridemic-waist index; LAP, Lipid Accumulation Product; FLI, Fatty Liver Index; GGT, Gamma Glutamyl transferase, HSI, Hepatic Steatosis Index; ALT, alanine aminotransaminase; AST, aspartate aminotransaminase; ZJU, Zhejian University Index; FPG, Fasting Plasma Glucose; FIB-4, Fibrosis-4; APRI, AST to Platelet Ratio Index; ULN, Upper Limit of Normal; NFS, NAFLD Fibrosis Score; T2D, Type 2 Diabetes; PNFI, Pediatric NAFLD Fibrosis Index; PNFS, Pediatric NAFLD Fibrosis Score; PMI, Pediatric Metabolic Index.
